# Bioenergetic modelling of a marine top predator's responses to changes in prey structure

**DOI:** 10.1002/ece3.11135

**Published:** 2024-03-24

**Authors:** Mariana P. Silva, Cláudia Oliveira, Rui Prieto, Mónica A. Silva, Leslie New, Sergi Pérez‐Jorge

**Affiliations:** ^1^ Institute of Marine Sciences – OKEANOS University of the Azores Horta Portugal; ^2^ Institute of Marine Research – IMAR Horta Portugal; ^3^ Department of Mathematics and Computer Science Ursinus College Collegeville Pennsylvania USA

**Keywords:** bioenergetics, energy, foraging success rate, marine mammal, modelling, physiological ecology, prey availability, prey size

## Abstract

Determining how animals allocate energy, and how external factors influence this allocation, is crucial to understand species' life history requirements and response to disturbance. This response is driven in part by individuals' energy balance, prey characteristics, foraging behaviour and energy required for essential functions. We developed a bioenergetic model to estimate minimum foraging success rate (FSR), that is, the lowest possible prey capture rate for individuals to obtain the minimum energy intake needed to meet daily metabolic requirements, for female sperm whale (*Physeter macrocephalus*). The model was based on whales' theoretical energetic requirements using foraging and prey characteristics from animal‐borne tags and stomach contents, respectively. We used this model to simulate two prey structure change scenarios: (1) decrease in mean prey size, thus lower prey energy content and (2) decrease in prey size variability, reducing the variability in prey energy content. We estimate the whales need minimum of ~14% FSR to meet their energetic requirements, and energy intake is more sensitive to energy content changes than a decrease in energy variability. To estimate vulnerability to prey structure changes, we evaluated the compensation level required to meet bioenergetic demands. Considering a minimum 14% FSR, whales would need to increase energy intake by 21% (5–35%) and 49% (27–67%) to compensate for a 15% and 30% decrease in energy content, respectively. For a 30% and 50% decrease in energy variability, whales would need to increase energy intake by 13% (0–23%) and 24% (10–35%) to meet energetic demands, respectively. Our model demonstrates how foraging and prey characteristics can be used to estimate impact of changing prey structure in top predator energetics, which can help inform bottom‐up effects on marine ecosystems. We showed the importance of considering different FSR in bioenergetics models, as it can have decisive implications on estimates of energy acquired and affect the conclusions about top predator's vulnerability to possible environmental fluctuations.

## Graphical abstract

Determining how animals allocate energy, and how external factors influence this allocation, is crucial to understand species' life history requirements and response to disturbance. Our manuscript presents a bioenergetic modelling approach to describe the daily energy acquisition and requirements of a marine top predator, based on prey characteristics and foraging data from stomach content and animal‐borne tags, respectively. The model is used to investigate the impact of changing prey structure on the energetics of sperm whales (*Physeter macrocephalus*), which consequently can help inform top‐down effects on marine ecosystems, but its basic principles are universal, and it could be parameterized to other marine top predator species.

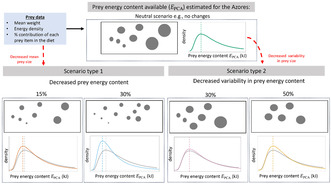

## 1 INTRODUCTION

Natural and human‐induced threats such as climate change, fisheries, industrial development, whale‐watching activities and pollution contribute directly and indirectly to the degradation and destruction of marine habitats (e.g. Boavida‐Portugal et al., 2022; Czapanskiy et al., 2021; Myers & Worm, 2003; Oliveira et al., 2022; Pirotta et al., 2019). These threats have been increasing over the years and predator populations can be affected by associated energetic costs due to food limitation and lost foraging opportunities caused by decreases in prey size and availability (Gallagher et al., 2021). To survive, animals need to balance the energy acquired from their prey and the energy allocated to essential metabolic processes such as maintenance, thermoregulation, movement, reproduction and growth (Booth, 2020; Booth et al., 2023). Stressor‐induced costs, depending on their severity, can impact this allocation and negatively affect individuals' reproduction and survival, making populations more vulnerable to environmental fluctuations and anthropogenic disturbance (Pirotta et al., 2019). Thus, it is necessary to predict how animals will respond to the stressors and their impact on the environment.

Bioenergetics models have been used to understand the effects of stressors, within the population consequences of disturbance (PCoD) framework (Gallagher et al., 2021; New et al., 2014; Pirotta et al., 2014). The PCoD framework aims to forecast plausible population‐level consequences following animals' exposure to stressors (Pirotta et al., 2018) using information on species' foraging patterns, life‐history traits and demographic parameters. Within PCoD, bioenergetic models are used to assess how changes in individuals' behaviour and physiology in response to disturbance might impact individuals' health and vital rates and, in turn, affect populations (Pirotta et al., 2018). However, the data necessary to parameterize these models, specifically regarding prey information, is often sparse, making it difficult to assess population‐level consequences (Booth et al., 2017, 2023). Additionally, most efforts have focused on assessing the influence of stressors in energy expended, and limited information exists on the effects on energy intake (Booth et al., 2023).

A key component to estimating animals' energy intake is foraging success as it expresses their ability to obtain energy from the environment (Blakeway et al., 2021) and is associated with animals' fitness (Hintz & Lonzarich, 2018) thus having repercussions on species' survival and reproductive success (Jeanniard‐Du‐dot et al., 2017). Individuals are considered successful in their environment when the energy obtained exceeds the energy expended to search, capture and handle prey, as well as to fulfil essential life functions (Booth, 2020; Jeanniard‐Du‐dot et al., 2017). Animals' maximisation and optimization of foraging success varies with multiple factors, such as resource distribution, group size, habitat, predation and competition (Hintz & Lonzarich, 2018). Direct observations of interactions between predator and prey are needed to help us estimate foraging success rate (FSR). However, this is logistically difficult and challenging, particularly for marine predators foraging underwater and at great depth (Miller, Johnson, & Tyack, 2004). Foraging success has been hypothesised to vary with behavioural factors such as dive type (e.g. benthic, epipelagic and mesopelagic dives; Blakeway et al., 2021) and dive metrics (e.g. bottom and dive duration; Viviant et al., 2014). For marine predators, such as cetaceans, empirical data on foraging success is scarce and it is often assumed that all feeding events capture prey successfully (Czapanskiy et al., 2021; Goldbogen et al., 2019; Wisniewska et al., 2016). This assumption is based on information from harbour porpoises (*Phocoena phocoena*), which have a high cost of living in perspective to other cetacean species (Spitz et al., 2018), making the species dependent on high foraging rates to support their metabolic requirements (>90% FSR estimated in Wisniewska et al., 2016). High FSR has also been assumed for deep divers, such as sperm whales (*Physeter macrocephalus*) (Czapanskiy et al., 2021; Goldbogen et al., 2019), which might not correspond to their low cost of living (Czapanskiy et al., 2021; Spitz et al., 2018), and could have implications on estimates of the species' overall energy intake. To determine a minimum FSR, bioenergetic modelling approaches need to incorporate estimates of species' energetic requirements and information on prey energy density. Animals' energetic requirements can be estimated using metabolic rates as a proxy (e.g. Kleiber, 1947) as it is the rate at which body consumes energy and is associated with animals' physiology and ecological parameters (Czapanskiy et al., 2021).

Using female sperm whales in the Azores as a case study, the aim of this work was to predict the energetic effect of changes in prey structure and provide insight into a top predator's responses to these changes. Like many top predators, sperm whales are essential to the integrity and stability of the marine ecosystems and their decline may have profound ecological and economic consequences (Morato et al., 2016; Ressurreição et al., 2022). Thus, understanding the impact of changing prey structure on the energetics of sperm whales, and top predators generally, is extremely important and can consequently help inform bottom‐up effects on marine ecosystems.

Cephalopods, the main prey of sperm whales (Clarke et al., 1993), are highly sensitive to environmental changes, which can impact their life history characteristics (growth and maturation rates), as well as distribution and abundance (Pierce et al., 2008). Previous experimental and modelling work has shown that cephalopods' growth would benefit from increased temperature (Doubleday et al., 2016). However, other studies suggest that increased temperature may also have a negative impact on their metabolism and body size (Jackson & Domeier, 2003; Melzner et al., 2006; Pörtner, 1995). Projected population dynamics of cephalopods under rising water temperatures also exhibit complex patterns and nonlinear responses, from cephalopod population getting extinct by 2070 to an exponential population growth (André et al., 2010). Additionally, these population models showed that cephalopods' populations structure is likely to change substantially under different climate change scenarios, resulting in decreased generation times and changes in the proportion of size classes (André et al., 2010). More specifically, some climate change scenarios predicted an increase in the proportion of smaller individuals or a population structure dominated by juveniles.

Sperm whales mainly feed on deep‐water cephalopods, which due to their low metabolic rates and limited mobility (Seibel et al., 1997), might be less capable to deal with environmental shifts compared to species that are more adaptable to these changes (e.g. teuthoids) (Hoving et al., 2014). Indeed, some studies have suggested that deep‐water cephalopods may be affected by environmental changes (e.g. increase in water temperature and hypoxia conditions) (Gilly et al., 2013; Rodhouse, 2013). However, there is still limited information on these cephalopods, most of which is based on analysis of sperm whale stomach contents from the whaling industry, limiting our ability to predict how species will respond to environmental changes (Hoving et al., 2014; Smith & Whitehead, 1993).

**FIGURE 1:**
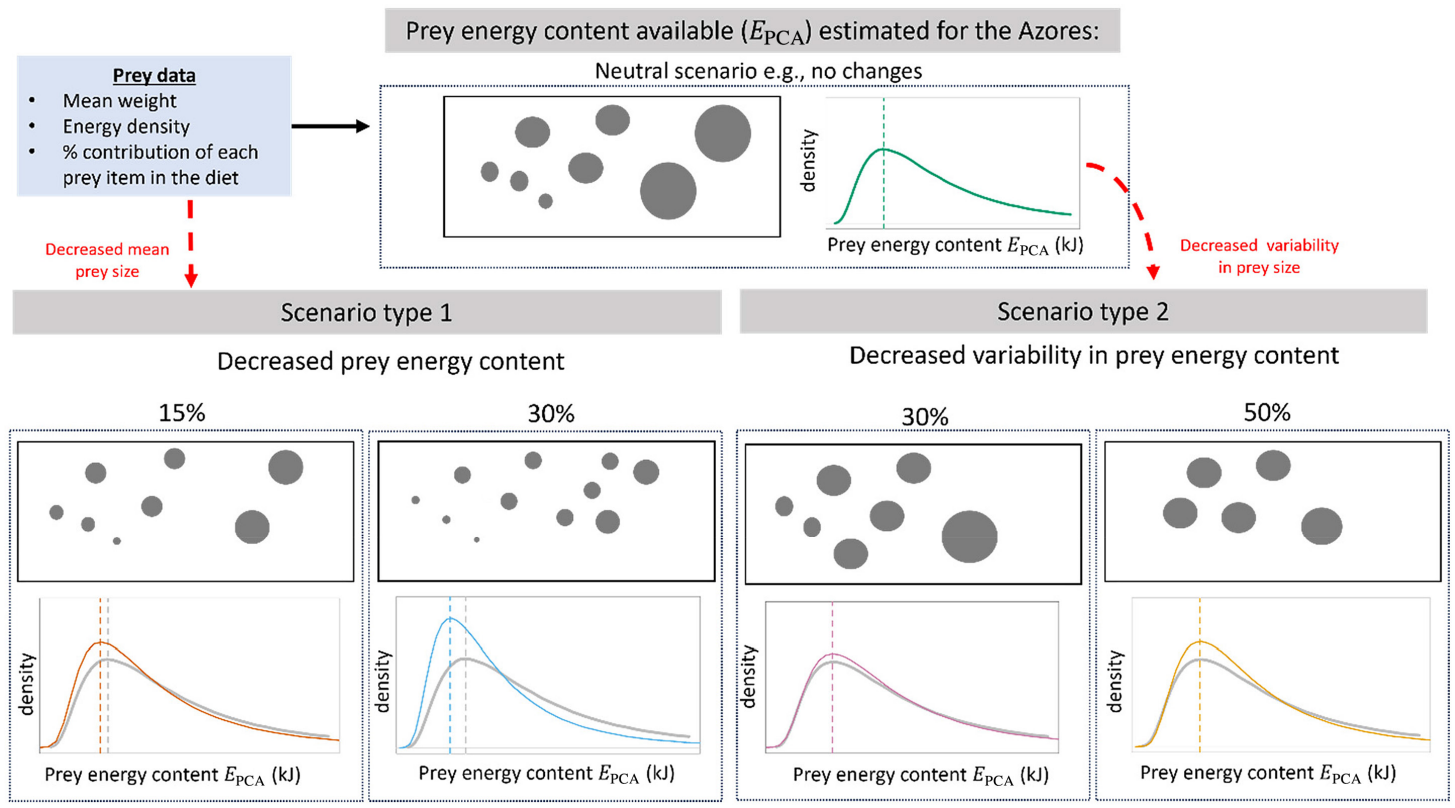
Modelling approach to simulate different prey structure scenarios: (1) decreased prey energy content (e.g. decreased mean prey size) and (2) decreased variability in prey energy content (e.g. changes in prey size variability); based on prey characteristics from analysis of stomach contents from historical whaling records (Clarke et al., 1993). The plots represent the potential energy acquired per PCA ($E_{\mathrm{PCA}}$) density curves using different log‐mean and variance values according to the scenarios simulated: neutral above in green, decreased prey size 15% and 30% below on the left side in orange and blue, respectively, decreased prey size variability 30% and 50% below on the right side in pink and gold, respectively. Dashed lines are the mode of each distribution. The dots are a visual representation of the potential energy available for each of the scenarios.

Based on the uncertainties surrounding cephalopods' response to environmental change, we carried out a modelling exercise simulating two potential prey structure scenarios to evaluate energy‐mediated responses of sperm whales to changes in prey resources (Figure 1). The first scenario modelled a decrease in mean prey size, and thus a lower prey energy content available per prey capture attempt (PCA). In the second scenario, we modelled a decrease in the variability of prey energetic content through a reduction of the prey size variability, as might occur as a result of changing prey structure, such as smaller juveniles dominating the population. These scenarios were implemented in a bioenergetic model developed using foraging data and prey characteristics from animal‐born tags and stomach content records, respectively, together with theoretical energetic requirements to estimate a minimum FSR. Finally, the sperm whales' vulnerability to changes in energy resources was evaluated using the ratio of energy requirements to energy acquired. This information was used to explore the level of compensatory energy intake required to continue to meet energetic needs under different environmental scenarios.

## 2 MATERIALS AND METHODS

### 2.1 Data collection and model description

The daily energy acquisition (Pa, kJ/day) was modelled as the product of PCA rate ($r_{\mathrm{PCA}}$, buzzes per hour) and the sum of the potential energy acquired per capture attempt ($E_{\mathrm{PCA}}$, kJ) within a day, considering different FSR and assimilation efficiencies (AE) (Equations 1 and 2).

(1) $$\mathrm{Pa}=\mathrm{AE}\times \sum_{h=1}^{24}\sum_{p}^{S_{r_{\mathrm{PCA}},h}}E_{\mathrm{PCA},p}$$

(2) $$S_{r_{\mathrm{PCA}}}=r_{\mathrm{PCA}}\times \mathrm{FSR}$$

where *p* is the number of PCAs, and $S_{r_{\mathrm{PCA}}}$ is the rate of successful PCA.

Female sperm whales' $r_{\mathrm{PCA}}$ was estimated based on acoustic data collected using high‐resolution multi‐sensor tags (DTAGs, Johnson & Tyack, 2003). Acoustic data were obtained from 11 individuals tagged in the Azores between 2018 and 2020 (Table S1) and were used to identify the number of foraging buzzes (i.e. short‐range fast echolocation clicks trains) which likely indicate PCA (Miller, Johnson, Tyack, & Terray, 2004). DTAG data were calibrated and analysed in Matlab 2007b and 2016b (Mathworks, Inc.) with custom tools developed specifically for these data‐loggers (https://www.soundtags.org/dtags/dtag‐toolbox/). For each whale, an $r_{\mathrm{PCA}}$ per hour was calculated. Based on the work of Miller et al. (2009), the first dive cycle or 1‐h of deployment was removed from the dataset to minimise potential effects foftagging on whale behaviour, such as shorter dive durations, reduced buzz rates or pitching movements in bottom phases. To estimate individuals' foraging activity within a day, $r_{\mathrm{PCA}}$ per hour were randomly sampled from the original DTAG data to fill a 24 h period per individual.

The $E_{\mathrm{PCA}}$ was modelled as a log‐normal distribution (Czapanskiy et al., 2021) to simulate patchily distributed prey (Benoit‐Bird et al., 2019; Pagel et al., 1991; Sims et al., 2008). The distribution for $E_{\mathrm{PCA}}$ was parameterized using sperm whales' prey weight ($W_{\text{prey}}$; g) and energy density data (ED; kJ/g) (Goldbogen et al., 2019) (Table S2). The mean and variance of log‐normal distribution of $E_{\mathrm{PCA}}$ were calculated from $\log\left(W_{\text{prey}}*\mathrm{ED}\right)$ and weighted by the proportional contribution of each prey item in the diet (%). Information on prey species was obtained from stomach content records of sperm whales caught during the whaling period (1981–1984) in the Azores (Clarke et al., 1993). When $W_{\text{prey}}$ was not available, size ranges were converted to weight values by applying a weight‐length relationship (Foskolos et al., 2020) (Equation S1).

Finally, the daily energy acquisition was obtained by summing Pa over 24 h. In addition, the metabolised energy was considered by exploring different AE previously reported for cetaceans: 74% for a small cetacean, the harbour porpoise (Yasui & Gaskin, 1986), 80% for large cetaceans (Booth et al., 2023; Lockyer, 2007) and 95% representing a best case‐scenario (Booth, 2020).

To account for differences between individuals and natural variability in energy acquisition (environmental stochasticity), Monte Carlo simulations were conducted to estimate Pa, obtaining a mean across all simulations that were used to make assumptions about the entire population (Table 1).

**TABLE 1:** Model parameters, their abbreviations, units and the values, equations or distributions from which parameter values are drawn, with references to literature source.

Parameter	Abbreviation	Unit	Distribution, value, equation	Reference
Foraging success rate	FSR	%	Gradually increased (0–100)	This study
Daily energy acquisition	Pa	kJ/day	$\mathrm{Pa}=\mathrm{AE}\times \sum_{h=1}^{24}\sum_{p}^{S_{r_{\mathrm{PCA}},h}}E_{\mathrm{PCA},p}$	This study, modified from Czapanskiy et al. (2021)
Assimilation efficiency	AE	%	Value, 74, 80 or 95	Lockyer (2007)
Prey capture attempt rate	$r_{\mathrm{PCA}}$	Buzzes per hour	Extrapolated from empirical data, see Table S1	This study
Rate of successful prey capture attempts	$S_{r_{\mathrm{PCA}}}$	Successful buzzes per hour	$S_{r_{\mathrm{PCA}}}=r_{\mathrm{PCA}}\times \mathrm{FSR}$	This study
Potential energy acquired per capture attempt	$E_{\mathrm{PCA}}$	kJ	Log‐normal distribution, Mean = 8.923264, SD = 0.7745222	This study estimated using prey data from Clarke et al. (1993) and energy density from Goldbogen et al. (2019)
Weight of prey	$W_{\text{prey}}$	g	For values, see Table S2	Clarke et al. (1993)
Energy density	ED	kJ/g	For values, see Table S2	Goldbogen et al. (2019)
Basal metabolic rate	BMR	kJ/day	$\mathrm{BMR}=293.1\times M^{0.75}$	Kleiber (1947)
Field metabolic rate	FMR	kJ/day	See Table S3	Kleiber (1947); Nagy et al. (1999); White and Seymour (2003); Savage et al. (2004); Kolokotrones et al. (2010); Spitz et al. (2018)
Average daily metabolic rate	ADMR	kJ/day	ADMR≈FMR=β*BMR	Spitz et al. (2018)
Species‐specific parameter accounting for activity costs	β	—	*β* = 2, 3 or 4 for species with low, medium and high cost of living, respectively	Spitz et al. (2012, 2018)
Proportion of compensation	*Y*	%	$Y=\frac{\text{ADMR}\;}{\mathrm{Pa}}-1$	This study

### 2.2 Energetic requirements: Metabolic rates

To understand individuals' energetic requirements (see below) and estimate minimum FSR, we estimated daily energetic requirements of sperm whales using metabolic rates (MR), the rate at which animals use their energy, as a proxy for their energetic needs. MR, such as basal metabolic rate (BMR, kJ/day) and field metabolic rate (FMR, kJ/day), are often used to estimate energetic requirements as they are associated with many phylogenetic, physiological and ecological parameters (Czapanskiy et al., 2021). BMR is a function of individual body mass and represents the body's requirements to accomplish its most basic life‐sustaining functions such as breathing and circulation (Spitz et al., 2012). FMR, also based on individual body mass, extends our understanding of animal's needs, as it includes information about the metabolic costs associated with physical and physiological activities, such as foraging, reproduction and thermoregulation (Farmer et al., 2018; Spitz et al., 2018). Measurements of body mass were not available for sperm whales in this study, thus, were obtained from length‐mass relationships. An average body length of 861 (±89) cm for female sperm whales in the Azores was obtained by photogrammetry measurements of eight individuals (unpublished data), and was within the ranges of female body lengths reported from whaling data (Clarke et al., 1993). The length measurements were converted to mean body weight using published equations (Lockyer, 1991; Equation S2).

There are a number of different equations used to calculate FMR for mammalian species (Kleiber, 1947; Kolokotrones et al., 2010; Nagy et al., 1999; Savage et al., 2004; Spitz et al., 2018; White & Seymour, 2003) (Table S3 and Equations S3–S9). Each approach results in distinct estimates of individuals' energetic requirements, thus influencing the results of energetic models (Table S4). After exploring multiple approaches to calculating MR, we decided to use the average daily metabolic requirement (ADMR, kJ/day) (Equation 4) estimated from a generic model of BMR (Kleiber, 1947) (Equation 3) (Spitz et al., 2018). We considered ADMR to be the most appropriate because it uses a generic model of basal metabolic rate and it includes a parameter (β) that accounts for the animal's cost of living (Spitz et al., 2018). This parameter, incorporates physical and physiological energetic costs (for example, cost of foraging and thermoregulation, not including pregnancy and lactation) and can take values of 2, 3 or 4 for species with low, medium and high cost of living, respectively (Spitz et al., 2018). For sperm whales, we used β=2, corresponding to a stealth species with slow swimming that consumes low‐quality prey (Spitz et al., 2012, 2018):

(3) $$\mathrm{BMR}=293.1\times M^{0.75}$$

(4) ADMR≈FMR=β×BMR

### 2.3 Prey structure scenarios

Cephalopods are believed to succeed under climate change conditions (Doubleday et al., 2016), particularly based on their life‐history characteristics (short‐lived, fast‐growing and semelparous) which makes them highly adaptable to environmental changes (Boavida‐Portugal et al., 2022; Pierce et al., 2008; Rodhouse, 2013; Wood & O'Dor, 2000). However, cephalopods have also been shown to be quite sensitive to warmer temperatures, leading to a decrease in their body size (Jackson & Domeier, 2003) and changes in their population structure (André et al., 2010). Based on these findings, it is possible that cephalopod's response to climate change could lead to changes in their size and population structure, affecting the energy available to the sperm whales. Thus, we modelled two different scenarios reflecting possible changes in sperm whales' prey structure by manipulating the modelled distribution of $E_{\mathrm{PCA}}$, particularly the mean and variance (Figure S2).

The first scenario sought to understand the energetic consequences of decreased prey size, and thus lower prey energetic content, to sperm whales. The baseline mean value for $E_{\mathrm{PCA}}$ was calculated from the mean $W_{\text{prey}}$ from historical diet records (Clarke et al., 1993) and was assumed to represent the current energy gained per PCA, and thus the average energetic content of sperm whales' prey. To simulate a moderate and worst‐case scenario we decreased the mean of the data by 15% and 30%, respectively, and left variance unchanged (Table S5). The second scenario sought to assess the energetic impact of changes in the distribution of prey energy content resulting from a decrease in prey size variability. In this scenario, we simulated a reduction in prey energy variability, representing shifts in prey use of the ocean or population structure, for example, population structure dominated by juveniles (André et al., 2010). These shifts may occur as a consequence of prey adapting to new climate conditions by changing their distribution, such as high energetic prey moving out of the whales' foraging grounds. For example, during El Niño events (i.e. increased temperatures and decreased ocean productivity), it was suggested that cephalopod stocks move offshore to find productive waters (Zeidberg et al., 2006). Specifically, we reduced the variability on the scale of the data while retaining a constant mode, which does result in a slight decrease in the mean value of $E_{\mathrm{PCA}}$ (Table S5). By maintaining a constant mode the most commonly encountered value of $E_{\mathrm{PCA}}$ did not increase, as it would have if the mean was held constant. The constant mode and reduced variance results in the most commonly available prey being consistent with that in the neutral scenario. Mimicking a biological situation in which the whales' most commonly encountered prey aggregations and prey items will remain the same, but in which they will no longer have access to extremely large or extremely small aggregations of prey or energetic prey items. Although we decrease variability, we are not simulating a truly homogeneous prey field because the probability of encountering prey is not constant, rather when prey patches are encountered, they are of a more consistent energetic value. For this scenario, we reduced the variance of the data by 30% and 50% of the baseline corresponding to assumed moderate and worst‐case scenarios, respectively (Figure S2). As there is no empirical data on the decrease of prey size in deep‐water cephalopods or changes in their prey structure, the scenarios were chosen to allow a clear relative comparison of the effects of reductions in mean prey size or prey size variability while remaining within the realm of biological plausibility. For more details on how the scenarios were created including the changed parameters please see ‘Prey structure scenarios’ section in Supporting Information.

### 2.4 Energetic compensation for a decrease in energy intake

To understand the energetic compensation required in response to a reduction in the mean or variance of energetic intake per PCA, a compensation ratio (*Y*), the ratio of energy required (ADMR) to energy acquired each day (Pa), was estimated for each of the four scenarios simulated (Equation 5). A positive value for *Y* represents changes in prey structure that have led to insufficient energy acquired to meet mean energy requirements and is assumed to result in a need for whales to increase their energy intake to compensate for the loss. In contrast, when *Y* is negative it represents a surplus of energy beyond what is required to meet daily life functions. Lastly, when *Y* is 0 whales are assumed to get the energy required per day:

(5) $$Y=\frac{\text{ADMR}\;}{\mathrm{Pa}}-1$$

All analyses were done using R software version 4.1.0 (R Core Team, 2021).

## 3 RESULTS

### 3.1 Baseline energy acquisition, requirements and minimum foraging success

**FIGURE 2:**
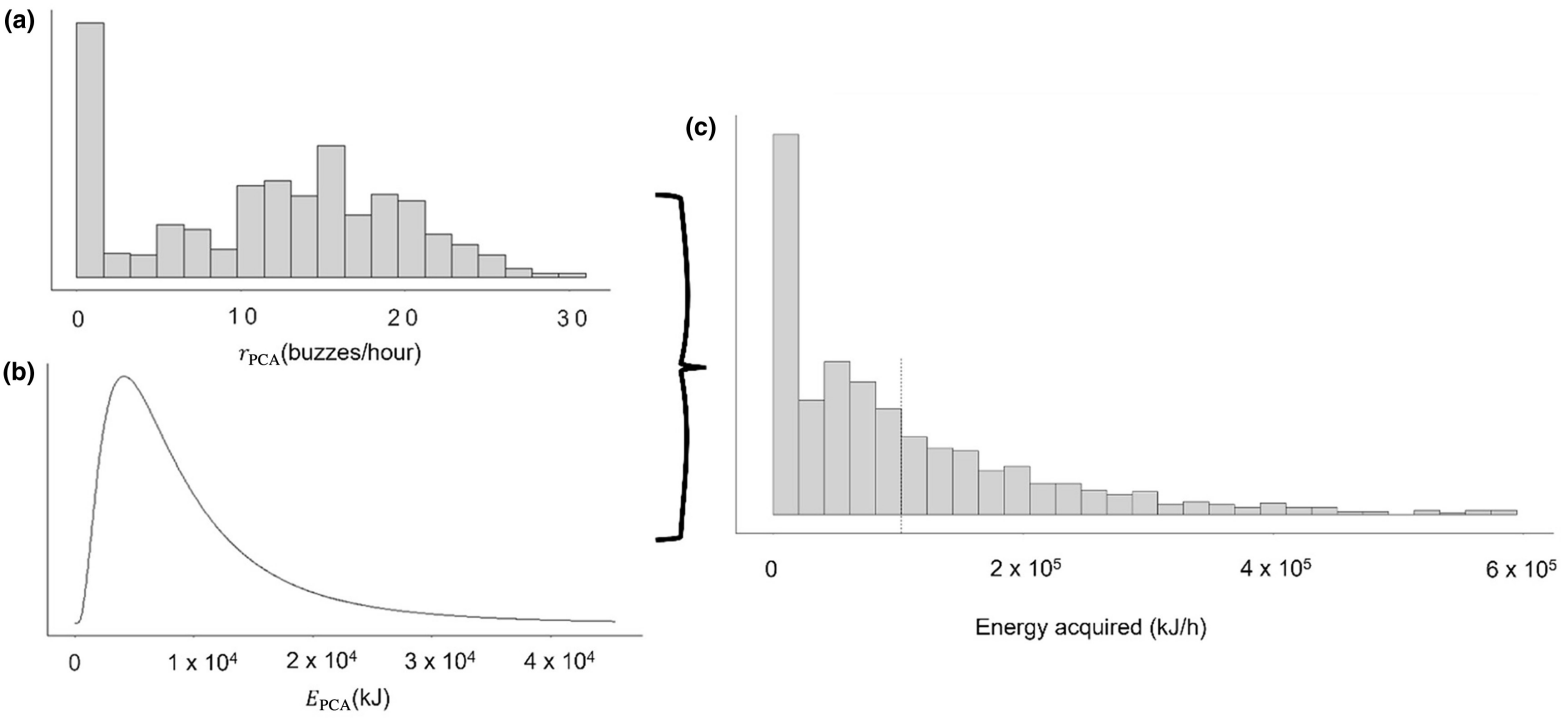
(a) Prey capture attempt per hour ($r_{\mathrm{PCA}}$), as the number of buzzes per hour calculated from the DTAG data. (b) Distribution of the potential energy available to sperm whales from prey in the area for each prey capture attempt ($E_{\mathrm{PCA}}$). (c) Example of baseline rate of energy acquired per hour as the product of $r_{\mathrm{PCA}}$ and $E_{\mathrm{PCA}}$. Mean energy acquired indicated with dashed line.

**FIGURE 3:**
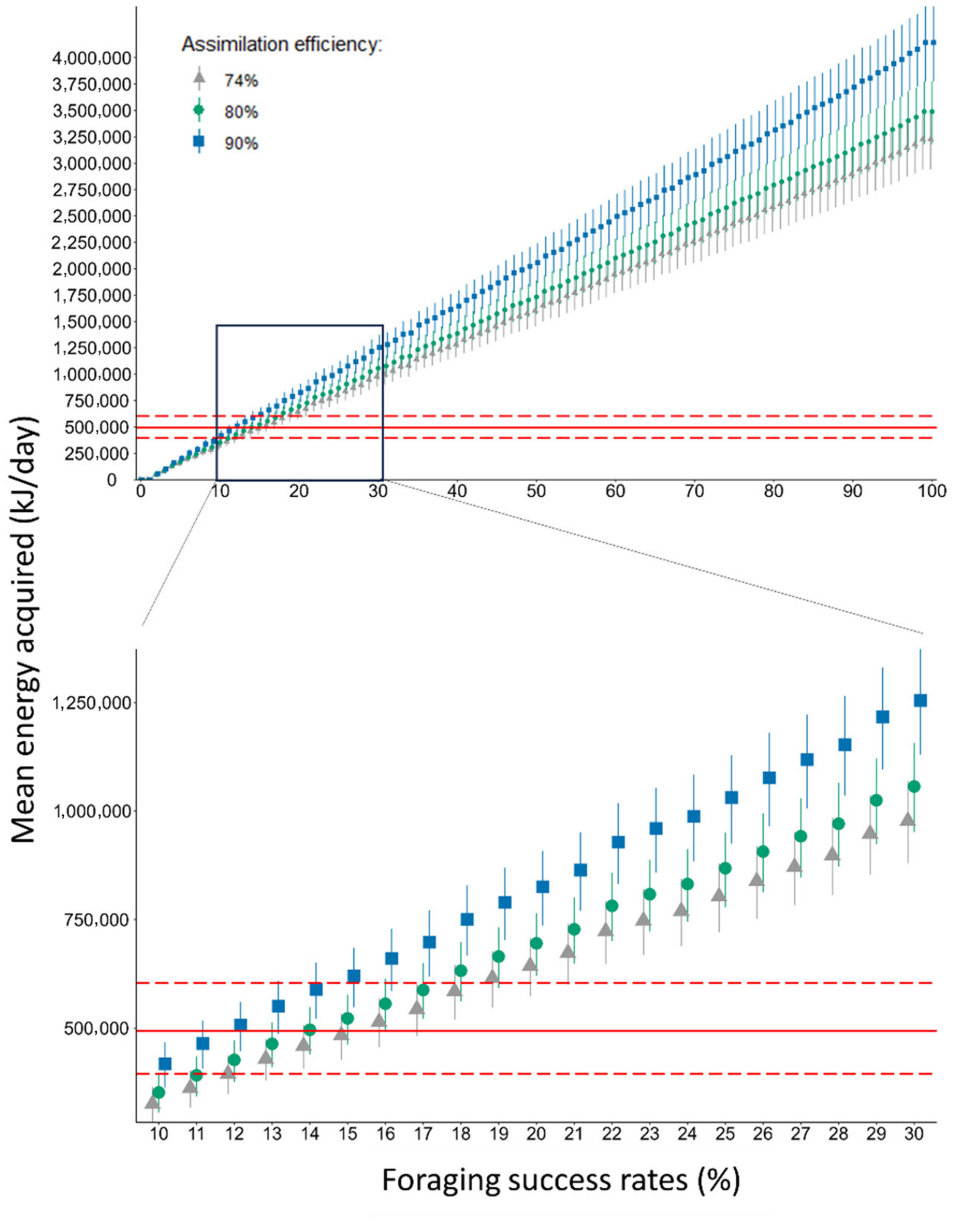
Plausible range of daily energy acquisition by sperm whales, as the product of potential energy per capture attempt, foraging success rate and assimilation efficiencies of 74% (grey triangles), 80% (green circles) and 95% (blue squares). Average daily metabolic requirement (ADMR = 493,539 kJ/day, solid red line) and minimum and maximum values of ADMR (394,425 and 604,091 kJ/day, dashed red lines) are also shown. Upper plot: foraging success rates varying from 0% to 100% in increments of 1%. Lower plot: zoom of upper plot with foraging success rates from 10% to 30% in increments of 1%.

A mean capture attempt rate $\left(r_{\mathrm{PCA}}\right)$ of 18 (Interquartile range, Q1–Q3: 10–25) events per hour (Table S1) was estimated for tagged sperm whales in the Azores. The mean prey energy content available ($E_{\mathrm{PCA}}$) estimated for the Azores was 10,129 kJ (SD = ±9183 kJ) per prey item (Figure 2b). The baseline rate of energy acquired per hour (Figure 2a) was obtained from the product of $r_{\mathrm{PCA}}$ and $E_{\mathrm{PCA}}$ (randomly paired) (Figure 2b,c). We estimated that sperm whales have an average daily metabolic requirement (ADMR) of 493,539 kJ/day (with a minimum and maximum of 394,425 and 604,091 kJ/day based on individuals' estimated body weight). When combined with the estimated distribution for mean energy acquired per day and including assimilation efficiencies (AE) of 74%, 80% and 95%, we identified a threshold FSR of approximately 14% (Figure 3) to meet ADMR. Observed results and patterns were similar across all three values of AE; therefore, we present only the results based on an AE of 80% (see Table S6, Figures S3–S6 for results using 74% and 95% AE).

### 3.2 Prey structure scenarios

**FIGURE 4:**
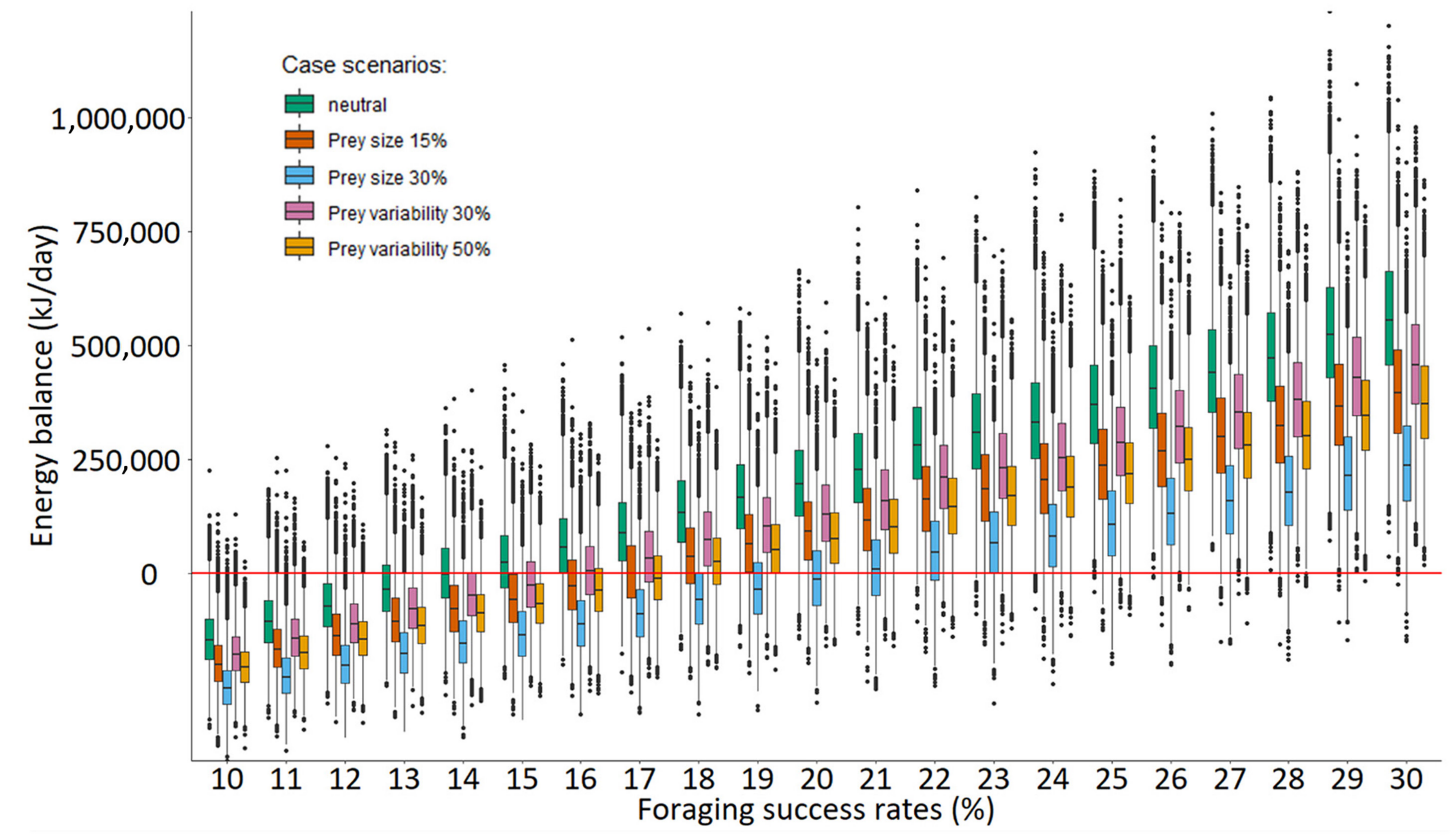
Energy balance per day is the difference between estimated energy acquisition per day relative to sperm whales' estimated energy requirements over foraging success rates and assimilation efficiency of 80%, for all simulated scenarios. Case scenarios represented by colours – green: neutral‐case (e.g. no changes); orange and blue: prey size moderate and worst with a decrease energetic content of 15% and 30%, respectively; pink and golden: prey size variability moderate and worst with a decrease of 30% and 50% of energy variability, respectively. Reference red dashed line: average daily metabolic requirement (ADMR, kJ/day).

In all scenarios exploring the effect of potential changes in prey structure, the energy acquired per day failed to reach the ADMR assuming a baseline FSR of 14%. The minimum FSR required to meet the minimum daily energy requirements was higher and varied between 17% and 21% (Figure 4) suggesting that under all prey structure scenarios, whales would need to compensate for a decreased energy intake. The decrease in energy intake was highest in scenarios including a decrease in prey size (lower prey energetic content) compared to a reduction in prey size variability (e.g. decrease variability on prey energy content) (Figure 4), implying that FSR is more sensitive to changes in average energy intake than variability in energy intake.

### 3.3 Understanding vulnerability to changes in prey structure

**FIGURE 5:**
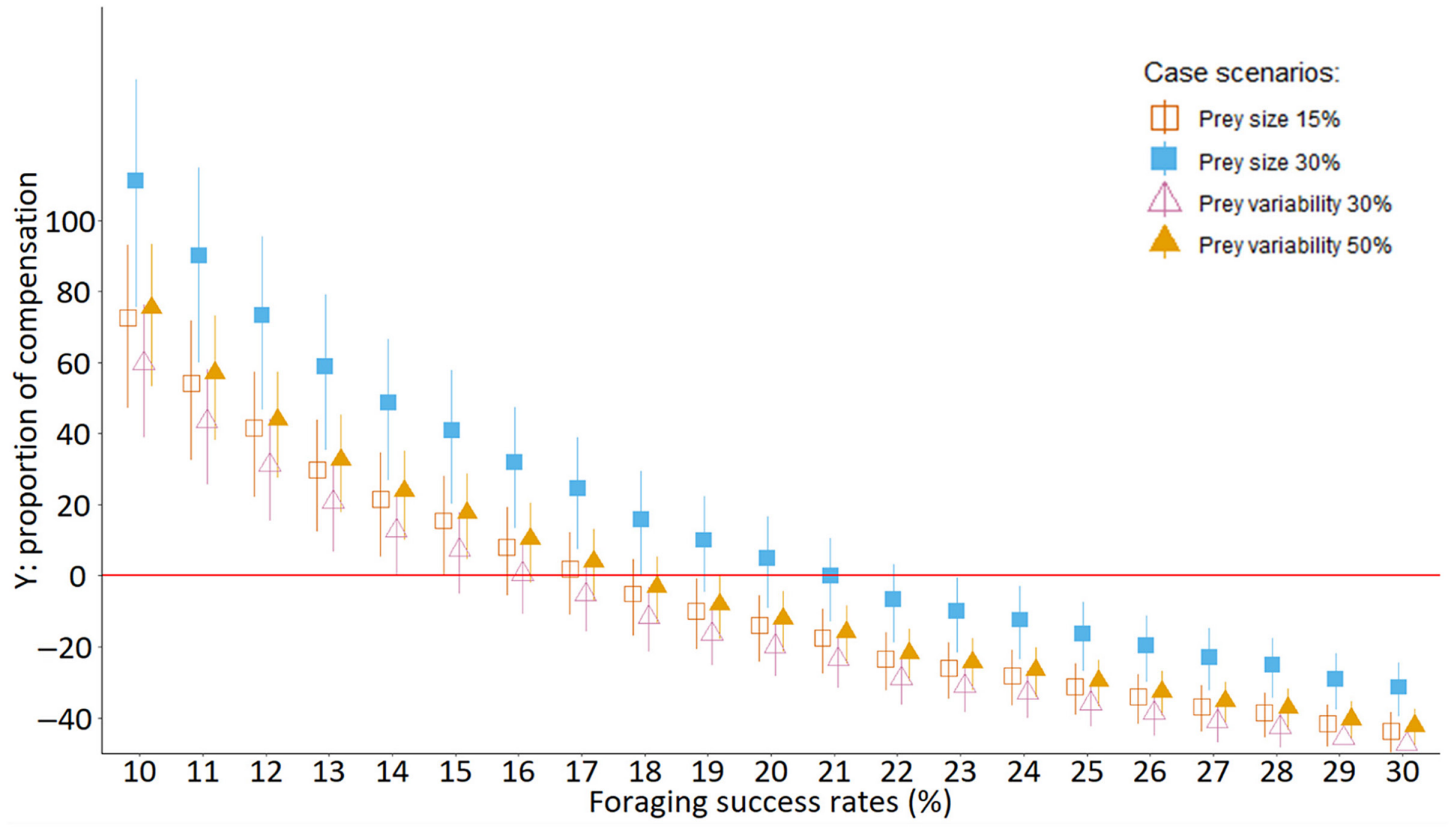
Compensation ratio (Y, the ratio of energy required to energy acquired per day) of sperm whales in all prey structure scenarios across different foraging success rates, using an assimilation efficiency of 80%. Positive Y: insufficient energy acquired to meet mean energetic demands. Negative Y: surplus of energy gained as a proportion of mean energetic demands. Y = 0: no compensation is needed. All four scenarios are represented, 15% decrease in prey size (empty orange squares), 30% decrease in prey size (filled blue squares), 30% decrease in prey size variability (empty purple triangles) and 50% decrease in prey size variability (filled gold triangles).

Comparing the prey structure scenarios with the baseline (i.e. at the minimum 14% FSR), whales would need to increase their energy intake by 21% (5–35%) and 49% (27–67%) to compensate for a decrease of 15% and 30% in prey size, respectively (Figure 5). Whereas for a decrease of 30% and 50% in the variability of the prey's size, whales would need to increase energy intake by 13% (0–23%) and 24% (10–35%) to meet their energetic requirements, respectively (Figure 5). Values are presented as mean (Q1–Q3).

## 4 DISCUSSION

Natural and anthropogenic disturbances can potentially affect the quantity of energy acquired by marine top predators, resulting in behavioural and physiological responses (Pirotta, 2022). To help determine the magnitude of the response that might be required, we built a bioenergetics model describing the daily energy acquisition and requirements of female sperm whales, which was based on prey characteristics and foraging data from stomach content and animal‐borne tags, respectively. Using the model, we identified a minimum required FSR and explored how that might change in response to changes in prey structure, which was modelled using energy acquired per capture attempt as a proxy. Prior to this, little work had been done to explicitly evaluate the required FSR of sperm whales, a fundamental prerequisite for modelling the bioenergetics of these marine top predators, or to assess the species' sensitivity to changes in prey structure.

### 4.1 Foraging success rate

Knowledge of FSR is crucial to predict how populations may respond to environmental changes (Speakman et al., 2020) because foraging is directly related to survival and fitness. Animals rely on successful food intake where energy gained exceeds energy expended (Booth, 2020; Jeanniard‐Du‐dot et al., 2017), while dealing with multiple and complex factors such as predation, competition and habitat quality (Bestley et al., 2010; Hintz & Lonzarich, 2018). Previous studies have assessed FSR of sperm whales using defecation rates. Since whales generally defecate before a dive, the defecation rate was defined as the proportion of slicks (the round patches on the water's surface that result from the force of a whale's tail as it dives) with defecation out of a minimum of 15 slicks checked and used as a proxy for feeding success (Jaquet & Whitehead, 1999; Rendell et al., 2004; Smith & Whitehead, 1993; Whitehead et al., 1989; Whitehead & Rendell, 2004). This approach assumes that deep‐diving mammals shut down physiological systems that are not immediately essential during a dive, and therefore that whales generally defecate at the surface (Whitehead et al., 1989). Sperm whale's FSR has also been observed to vary with movement patterns, where groups that frequently changed course had greater FSR (e.g. 8%–32%) compared to groups with more directed movement (e.g. 0%–3.6%), suggesting that movement patterns are influenced by the distribution of prey patches and consequently regulate animals' FSR (Jaquet & Whitehead, 1999). Moreover, sperm whales' FSR has been reported to be negatively correlated with sea‐surface temperatures, where colder upwelling and warmer waters are associated with high and low FSR, respectively (Rendell et al., 2004; Smith & Whitehead, 1993; Whitehead et al., 1989; Whitehead & Rendell, 2004). This correlation is, however, likely due to changes in prey and patch distribution as a response to water temperature, rather than with temperature per se. Although these previous studies have provided valuable insights into sperm whales FSR, information on how this correlates with animals' energetic needs is still scarce (Farmer et al., 2018), providing the impetus for our study. Our results indicate that a minimum FSR of 14% is necessary for female sperm whales to meet their average daily metabolic requirements (ADMR). This value represents the minimum success rate required to meet calculated energetic needs, however, the actual success rate from tagged whales remains unknown. These requirements consider animals' body weight as well as the energy required to sustain basic life functions and other physiological and physical activities for example breathing, thermoregulation, the cost of foraging, etc. (Czapanskiy et al., 2021; Farmer et al., 2018; Spitz et al., 2012, 2018). Direct measurements of field metabolic rates are difficult to obtain in marine mammals and standard methods cannot be applied to most large whales. In our study, we used different body‐mass relationship equations to calculate metabolic rate which resulted in distinct and very different assumptions of FSR (see Table S4, Figure 1 from Supplementary Materials for more details). These varying assumptions will influence the results of the bioenergetics models in which they are used, especially those intended to estimate the prey biomass consumed. These uncertainties underscore the need for better empirical estimates of energetics of large whales (Noren & Rosen, 2023). Our results indicate that sperm whales can survive, exploit their habitat and obtain enough energy to fulfil their energetic demands with lower FSR than has been previously assumed in bioenergetics studies. Our results are well below the FSR above 90% reported for harbour porpoises (Booth, 2020; Wisniewska et al., 2016), which is the basis of FSR assumptions for many cetacean species, including sperm whales (Czapanskiy et al., 2021; Goldbogen et al., 2019). However, given our results, and the fact that they are in line with the estimates of sperm whale's FSR using defecation rates as a proxy (Czapanskiy et al., 2021; Goldbogen et al., 2019; Jaquet & Whitehead, 1999), it is probable that this species has a greater plasticity in the required FSR to meet their minimum energetic demands than has traditionally been assumed (Czapanskiy et al., 2021; Goldbogen et al., 2019). Additionally, FSR likely depends on target prey, their size, behaviour and swimming abilities, and this will in turn vary with foraging depth and across regions (Bi & Zhu, 2018; Oliveira, 2014; Pérez‐Jorge et al., 2023; Visser et al., 2021).

### 4.2 Vulnerability to changes in prey structure

A probable effect of climate change and increased exploitation of the marine environment is a change in prey structure, and thus in the energy available to top predators. This change could take the form of shifts in prey distribution, size or species composition (where whales shift to feed on less energetically valuable species) (e.g. Gallagher et al., 2021; Nøttestad et al., 2015). In our scenarios, we investigated how potential changes in prey structure might translate to changes in energy intake.

Changes in mean prey size (lower prey energy content) and prey size variability (reduced prey energy content variability) were found to impact sperm whale's energy acquisition, where the magnitude of the effect varied with the FSR. Energy acquisition was more sensitive to changes in mean prey size than to prey size variability. As a result, the minimum FSR required to meet energetic demands was higher in scenarios representing a reduction of mean prey size than prey size variability. Consequently, a reduction of prey size would require greater compensation to reach ADMR. That is, a 15% and 30% reduction of prey size resulted in a minimum FSR required of 17% and 21%, respectively. In these scenarios, a compensation level of 21% (5–35%) and 49% (27–67%) on energy intake would be required to meet their ADMR. Whereas reducing prey size variability, by 30% or 50%, a minimum foraging success of 16% and 18% was required, respectively. In these scenarios, whales require a compensation level of energy intake of 13% (0–23%) and 24% (10–35), to meet their energetic needs. These compensation levels are smaller compared to scenarios including decrease in prey size. Changes in prey abundance and characteristics influence energetic costs forcing animals to compensate due to increased foraging effort to obtain their baseline energy (Gallagher et al., 2021; Goundie et al., 2015). Estimates of energy intake for other marine top predators such as harbour seal (*Phoca vitulina*) and harbour porpoises (*Phocoena phocoena*) have also been reported to be most strongly affected by variations in prey size and its energetic content (Booth, 2020; Bowen et al., 2002; Gallagher et al., 2021). Since these predators feed mostly on fish and considering the exponential relationship between fish length and mass, small changes in prey size are expected to have a great impact on total energy availability and profitability (Gallagher et al., 2021). However, sperm whales' diet is composed mostly of cephalopods, and there is little information available to infer the relationship between prey size and profitability (Jereb & Roper, 2010). Nevertheless, our results reveal a broad range of potential outcomes should this relationship remain consistent and demonstrate that the energetic content of prey plays an important role in determining minimum FSR requirements and the overall energy intake of sperm whales.

Results from scenarios including a shift in the variability of prey energy content (e.g. prey size variability) showed a lower effect on energy acquisition, and thus on the FSR needed to meet minimum energetic requirements. This result was not unexpected, given the structure of the model, which retains a constant average energy intake. However, it is interesting that less variable, and therefore more consistent, energy intake did not allow sperm whales to meet their ADMR, implying that the occasional occurrence of high calorific feeding events, perhaps due to unusual concentrations of prey or large individual prey items, is an important component of sperm whales' energy balance. Top predators' ability to encounter dense patches of prey has been reported to increase foraging efficiency and consequently, energy intake (Benoit‐Bird et al., 2013; Goldbogen et al., 2011, 2019) offsetting mortality and even increasing reproductive success (Gallagher et al., 2021). Undeniably, sperm whale's diving capacity allows them to invest time and energy (Goldbogen et al., 2019) in prolonged dives where they can exploit deeper habitats potentially with less mobile and more abundant prey (Benoit‐Bird et al., 2016; Fais et al., 2016; White et al., 2007). Thus, it is possible that encounters with dense prey patches provide whales with the opportunity to compensate for the decreased optimal energy intake through an increase of PCA or foraging dives per day. However, these compensatory behaviours would increase their energy expenditure. Future research should focus on estimating such compensatory mechanics through the development of an agent‐based model (Gallagher et al., 2021). In addition, these results also offer valuable insights into the dependence on the variability in prey and could indicate the importance of conserving variation in prey species for marine mammal management (Pettorelli et al., 2015).

Our work can be used within the PCoD framework (Pirotta et al., 2018) as it makes a bridge between a stressor (environmental change) and health (energy budget), and addresses existing data gaps. One major challenge within the PCoD framework is the lack of data to parametrize bioenergetic models (McHuron et al., 2022; Pirotta, 2022). These gaps exist not only because calculating foraging and movement energetics of cetaceans is challenging and direct measurements are limited to small or medium‐sized animals (Czapanskiy et al., 2021), but also because there is insufficient knowledge on the ecology of most prey species, especially those without commercial value or are logistically difficult to sample. While our model has been parameterized for sperm whales, its basic principles are universal enabling it to be applied to other species to predict their energy‐mediated responses to possible environmental changes. Moreover, our work explores FSR in a distinctive way, emphasising the role it plays in bioenergetic models. We suggest that future bioenergetic models consider different FSR, as this parameter can have decisive implications on the estimates of the energy acquired by individuals and consequently affect the conclusions that can be drawn about top predator's vulnerability to environmental shifts.

## AUTHOR CONTRIBUTIONS

**Mariana P. Silva:** Data curation (equal); formal analysis (equal); methodology (equal); visualization (lead); writing – original draft (lead); writing – review and editing (lead). **Cláudia Oliveira:** Data curation (equal); investigation (equal); writing – review and editing (equal). **Rui Prieto:** Funding acquisition (equal); investigation (lead); project administration (equal); writing – review and editing (supporting). **Mónica A. Silva:** Conceptualization (equal); funding acquisition (equal); investigation (equal); project administration (equal); writing – review and editing (equal). **Leslie New:** Formal analysis (equal); methodology (supporting); supervision (equal); writing – review and editing (equal). **Sergi Pérez‐Jorge:** Conceptualization (equal); data curation (equal); formal analysis (equal); investigation (equal); methodology (equal); supervision (lead); writing – original draft (supporting); writing – review and editing (equal).

## FUNDING INFORMATION

The research was supported by the Portuguese Science & Technology Foundation (FCT), the Azorean Science & Technology Fund (FRCT), and the EU through research projects WATCH IT_Acores‐01‐0145‐FEDER‐000057, META_FA_06_2017_017, TRACE‐PTDC/MAR/74071/2006, MAPCET‐M2.1.2/F/012/2011, FCT‐IF/00943/2013/CP1199/CT0001, SUMMER‐H2020 GA 817806 (FEDER, COMPETE, QREN, POPH, ESF, Portuguese Ministry for Science and Education, AZORES2020). MPS was supported by a PhD grant from Fundo Regional da Ciência e Tecnologia (FCRT; M3.1.a/F/028/2020), SPJ and MAS were supported by SUMMER‐H2020, GA 817806, CO was supported by WATCH IT (Acores‐01‐0145‐FEDER‐000057), tenders with SRMCT/DRAM under project RAGES (GA 110661/2018/794607/SUB/ENV.C.2) and INTERTAGUA (MAC2/1.1a/385), and with the University of St. Andrews under project ACCURATE (ONR‐ID314_02‐14‐2019_0852–30). MAS and RP are co‐financed by AZORES2020, through the EU Fund 01‐0145‐FEDER‐000140 “MarAZ Researchers: Consolidate a body of researchers in Marine Sciences in the Azores”. Okeanos is funded by FCT – Foundation for Science and Technology under the project UIDB/05634/2020 and UIDP/05634/2020, and through the Regional Government of the Azores through the initiative to support the Research Centers of the University of the Azores and through the project M1.1.A/REEQ.CIENTÍFICO UI&D/2021/010.

## CONFLICT OF INTEREST STATEMENT

The authors declare there are no conflicts of interest.

## Supporting information


Appendix S1.

Appendix S2.

## Data Availability

Data will be deposited at the World Data Center PANGAEA. The modelling code will be available upon request. Nevertheless, data and metadata were submitted as Appendix S2 for review and publication.
