# Supplementary material for: Bioenergetic modelling of a marine top predator's responses to changes in prey structure
Source: Ecol Evol. 2024 Mar 24;14(3):e11135. doi: 10.1002/ece3.11135 (PMC10961477; doi:10.1002/ece3.11135)
Supplement: Supplementary file 1 — Appendix S1. [file ECE3-14-e11135-s001.docx]

*The following supporting information accompanies the article*

**Bioenergetic modelling of a marine top predator’s responses to changes in prey structure**

Mariana P. Silva^*^, Cláudia Oliveira, Rui Prieto, Mónica A. Silva, Leslie New, Sergi Pérez-Jorge

***Corresponding author:** Mariana P. Silva,

E-mail: [marianafpsilva@outlook.com](mailto:marianafpsilva@outlook.com)

# Supporting Information

## Data

The bioenergetic model developed in this study uses the following data: foraging buzzes from DTAGS deployed on sperm whales in the Azores (Table S1) and prey information (Table S2): diet composition, species’ weight (g), and correspondent proportion of sperm whales' diet obtained from Clarke et al., (1993) and energy density (kJ/g) from Goldbogen et al. (2019). The model also estimates energy requirements using different body-mass relationship equations from Kleiber (1947), Nagy et al. (1999), White & Seymour (2003), Kolokotrones et al. (2010) and Spitz et al. (2018) (Table S3).

For tagged sperm whales, we calculated a mean capture attempt rate ${(r}_{PCA})$ of 18 (Interquartile range, Q1-Q3: 10-25) events per hour. This value was calculated in relation to the time of dives performed (e.g., foraging data duration) (Table S1). In the model, to estimate individuals foraging activity within a day, we used equation 1 and randomly sampled $r_{PCA}$ from this original DTAG data to fill a 24h period per individual.

Table S1. Foraging data used in this study from DTAG deployed on sperm whales (n=11) in the Azores archipelago (years 2018-2020).

| **Individual ID** | **Tag duration (h)** | **Foraging data duration (h)** | **Foraging dives (n)** | **Feeding events (n)** |
| --- | --- | --- | --- | --- |
| 2 | 5 | 1 | 1 | 16 |
| 3 | 80 | 2 | 3 | 63 |
| 4 | 23 | 17 | 17 | 340 |
| 5 | 9 | 5 | 5 | 110 |
| 6 | 7 | 5 | 5 | 67 |
| 7 | 25 | 18 | 19 | 188 |
| 8 | 13 | 9 | 8 | 78 |
| 9 | 19 | 14 | 14 | 377 |
| 10 | 5 | 1 | 1 | 13 |
| 12 | 14 | 5 | 5 | 87 |
| 13 | 17 | 8 | 16 | 167 |

Table S2. Diet composition, species’ weight (minimum, maximum, and mean, in g) and correspondent proportion in sperm whales' diet from the Azores region, obtained from Clarke et al. (1993) . Energy density (kJ/g) obtained from Goldbogen et al. (2019).

| **Family** | **Species** | **Weight (g)** | | | **Energy density (kJ/g)** | **Total energy (kJ)** | **Proportion diet (%)** |
| --- | --- | --- | --- | --- | --- | --- | --- |
|  |  | **min** | **max** | **mean** |  |  |  |
| Alloposidae | *Haliphron atlanticus* | 109 | 1371 | 642 | 2.31 | 251.79 | 0.74 |
| Ancistrocheiridae | *Ancistrocheirus lesueurii* | 273 | 1357 | 728 | 2.31 | 630.63 | 0.56 |
| Architeuthidae | *Architeuthis dux* | 221 | 107887 | 23627 | 2.65 | 585.65 | 12.1 |
| Chiroteuthidae | *Chiroteuthis joubini* | 33 | 216 | 51 | 2.31 | 76.23 | 0 |
| Chiroteuthidae | *Chiroteuthis spB* | 33 | 216 | 66 | 2.31 | 76.23 | 0.02 |
| Chiroteuthidae | *Chiroteuthis spd* | 33 | 216 | 141 | 2.31 | 76.23 | 0.04 |
| Chiroteuthidae | *Chiroteuthis veranyi* | 33 | 216 | 139 | 2.31 | 76.23 | 0.02 |
| Cranchiidae | *Megalocranchia spA* | 81 | 656 | 316 | 1.69 | 136.89 | 0.4 |
| Cranchiidae | *Megalocranchia spG* | 289 | 3988 | 1138 | 1.69 | 488.41 | 0.12 |
| Cranchiidae | *Teuthowenia maculata* | 67 | 337 | 203 | 1.69 | 113.23 | 0.4 |
| Cranchiidae | *Teuthowenia megalops* | 78 | 302 | 205 | 1.69 | 131.82 | 0.77 |
| Cycloteuthidae | *Cycloteuthis akimushkini* | 225 | 1530 | 862 | 2.31 | 519.75 | 0.5 |
| Cycloteuthidae | *Discoteuthis lacinosa* | 131 | 417 | 278 | 2.31 | 302.61 | 1.36 |
| Gonatidae | *Gonatus steenstrupi* | 41 | 124 | 78 | 3.78 | 154.98 | 0.11 |
| Histioteuthidae | *Histioteuthis bonnellii bonnellii* | 82 | 1111 | 411 | 2.65 | 217.3 | 28.16 |
| Histioteuthidae | *Histioteuthis bonnellii corpuscula* | 48 | 151 | 123 | 2.65 | 127.2 | 0.01 |
| Histioteuthidae | *Histioteuthis celetaria* | 120 | 425 | 239 | 2.65 | 318 | 0.39 |
| Histioteuthidae | *Histioteuthis meleagroteuthis* | 60 | 453 | 267 | 2.65 | 159 | 0.06 |
| Histioteuthidae | *Histioteuthis miranda* | 246 | 836 | 490 | 2.65 | 651.9 | 0.59 |
| Histioteuthidae | *Histioteuthis reversa* | 37 | 100 | 65 | 2.65 | 98.05 | 0.01 |
| Histioteuthidae | *Stigmatoteuthis arcturi* | 213 | 687 | 465 | 2.65 | 564.45 | 3.48 |
| Lepidoteuthidae | *Lepidoteuthis grimaldii* | 260 | 11867 | 2286 | 4.01 | 1042.6 | 4.53 |
| Mastigoteuthidae | *Mastigoteuthis spA* | 85 | 325 | 154 | 1.82 | 154.7 | 0.05 |
| Octopodidae | *Octopodid* | 3238 | 3238 | 321 | 3.77 | 12207.26 | 0 |
| Octopoteuthidae | *Octopeuthis spG* | 468 | 1794 | 1060 | 3.08 | 1441.44 | 0.46 |
| Octopoteuthidae | *Octopoteuthis rugosa* | 132 | 553 | 344 | 3.08 | 406.56 | 0.48 |
| Octopoteuthidae | *Taningia danae* | 1028 | 39603 | 8533 | 3.08 | 3166.24 | 38.77 |
| Ommastrephidae | *Ommastrephes bartramii* | 108 | 14540 | 6367 | 4.01 | 433.08 | 0.15 |
| Ommastrephidae | *Todarodes sagittatus* | 435 | 6732 | 2538 | 4.01 | 1744.35 | 3.21 |
| Onychoteuthidae | *Onychoteuthis banksi* | 89 | 2642 | 135 | 4.53 | 403.17 | 0.01 |
| Onychoteuthidae | *Onychoteuthis borealijaponica* | 88 | 2642 | 873 | 4.53 | 398.64 | 0.36 |
| Pholidoteuthidae | *Pholidoteuthis massyae* | 188 | 2675 | 1708 | 3.78 | 710.64 | 2.14 |
| Vampyroteuthidae | *Vampyroteuthis infernalis* | 453 | 1264 | 643 | 2.31 | 1046.43 | 0.01 |

When prey’s wet mass (g) values were not available, size ranges (values of total, dorsal mantle length (DML), or lower hood/rostral lengths (LHL/LRL)) were converted to weight content value (*TW*) by applying a weight-length relationship (Foskolos et al., 2020). The following equation obtained from Foskolos et al. (2020) for *Octopus vulgaris* was used to estimate a minimum and maximum wet mass for Octopodidae.

$TW=6.1718\times LHL^{3.03}$ (S1)

## Energy requirements: Metabolic rates

Metabolic rates (*MR*) were used to calculate potential energy requirements and were estimated using different length-mass relationship methods (Table S3). To inform equations the predictive formula S2 for sperm whales’ whole body weight ($W_{body}$, in metric tons) from length ($L_{body}$, in m) reported in Lockyer (1991) was used. Here, an average body length of 861 (±89) cm for female sperm whales in the Azores region, obtained by photogrammetry measurements of eight individuals (unpublished data) was used.

$W_{body}=0.0218\times(L_{body})^{2.74}$ (S2)

The use of different body-mass relationship equations (Table S3) resulted in distinct, and very different, assumptions of threshold foraging success rate (Table S4, Fig. S1). Using scaling relationships allowing a curvature in a log-log space (Nagy et al., 1999) we estimated a threshold foraging success of approximately 17%. Using a quadratic term (Kolokotrones et al., 2010) and the average daily metabolic requirement (ADMR, Spitz et al., 2018) we estimated a threshold foraging success rate of approximately 14%. Whereas, with a quarter power scaling$, \frac{3}{4}$, following Kleiber (1947) and Savage et al. (2004) formulas, a 8% and 6% foraging success rate would be enough to meet whales’ requirements. Finally, using an allometric exponent of $\frac{2}{3}$ (White & Seymour, 2003) a minimum 4% would suffice. Considering the limitations of the different body-mass relationship methods, whales’ average daily metabolic requirement (ADMR = 493539 kJ/day) was used as a reference for energetic requirements (Spitz et al. 2018) in this study thus, assuming that sperm whales need approximately a 14% minimum foraging success to meet their needs (Fig. S1). The other scaling equations were not considered further because: 1) data used to inform the MR were biased for values of small mammals (White & Seymour, 2003), 2) the use of an allometric exponent of 2/3, which reduces estimates of metabolic rates when extrapolating for large animals (White & Seymour, 2003), or 3) a quadratic term implying very small and very large mammals have elevated MR (Kolokotrones et al., 2010).

Table S3. The various body-mass energy relationships used to estimate metabolic rates of sperm whales.

| **Reference** | **Equation** | | |
| --- | --- | --- | --- |
| Kleiber (1947) | $FMR=70\times W^{0.75}\times kcal day to$ $W$ | (S3) | W body mass in kg.  “Kcal day to W” is a conversion factor = 0.04843.  70 kcal: under standard conditions fasting homeotherms produce daily an average of about 70 kcal of heat per kg ^¾^. |
| Nagy et al. (1999) | $FMR=4.82\times W^{0.734}\times kJ day to W$ | (S4) | W body mass in g.  “kJ day to W” is a conversion factor = 0.01157.  4.82 is the normalization constant for all mammals. |
| White & Seymour (2003) | $BMR=4.34\times W^{0.67}\times mlO2 hr to W$ | (S5) | W body mass in g.  “mlO2 hr to W” is a conversion factor = 20.1/3600 (assuming 20.1 J per ml O2).  4.34 is the normalization constant. |
| Savage et al. (2004) | $BMR={10}^{(-1.739+0.737\times log10(W))}$ | (S6) | W body mass in g. |
| Kolokotrones et al. (2010) | $BMR={10}^{(-1.5078+0.5400\times log10(W)+0.0322\times log10(W)^{2})}$ | (S7) | W body mass in g. |
| Spitz et al. (2018) | $BMR=293.1\times M^{0.75}$ | (S8) | BMR is a function of individuals’ body mass (M in kg) (Kleiber, 1947). |
|  | $ADMR\approx FMR=\beta\times BMR$ | (S9) | AMDR is the average daily metabolic requirement (kJ/day).  FMR is the field metabolic rate (kJ/day).  β is a species-specific parameter accounting for activity costs and in this case (for sperm whales) is equal to 2, correspondent to species with low cost of living (Spitz et al., 2018). |


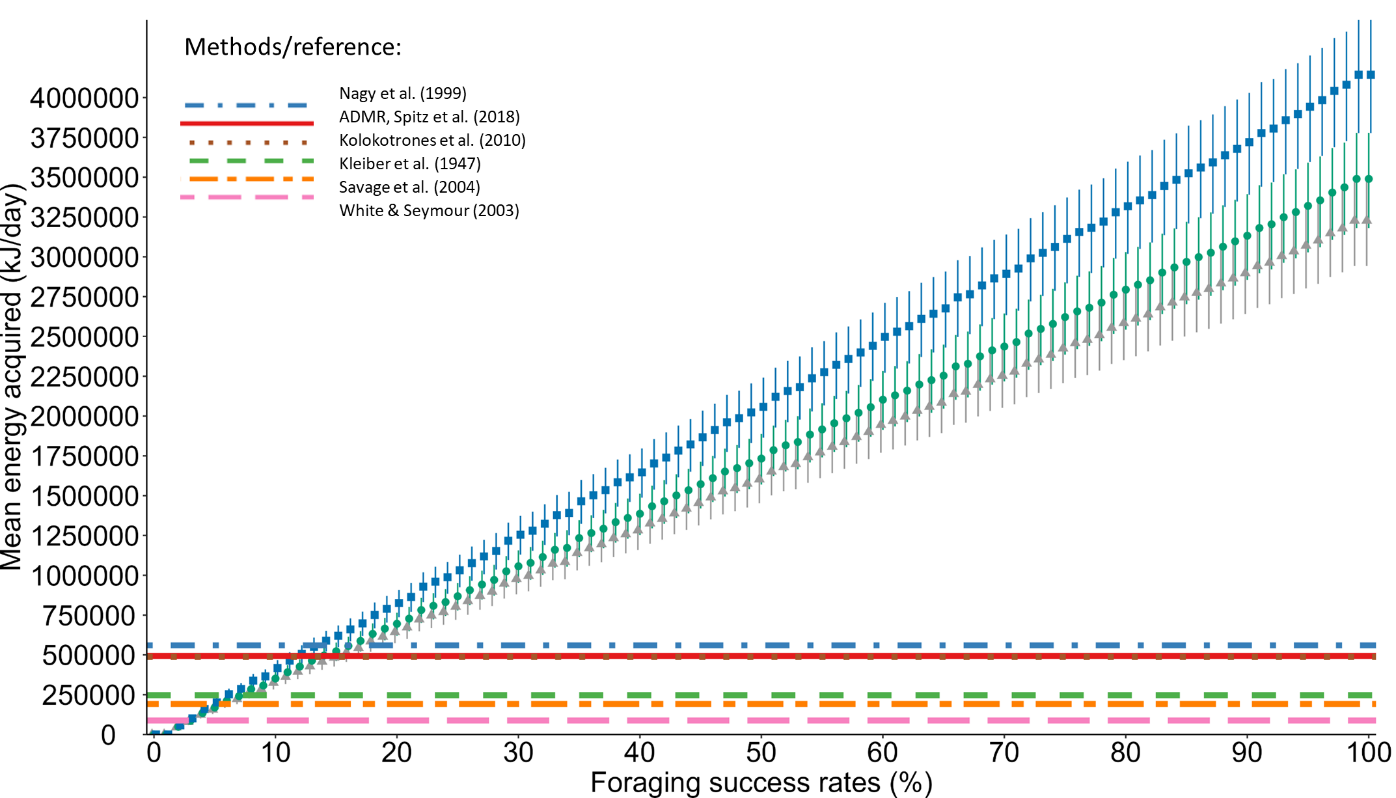


Figure S1. Plausible range of daily energy acquisition by sperm whales, as the product of potential energy per capture attempt, foraging success rate and assimilation efficiencies of 74% (grey triangle), 80% (green circle) and 95% (blue square). Interception lines are the mean levels of energy requirements estimated from different body-mass energy relationships to calculate metabolic rates (Kleiber, 1947; Nagy et al., 1999; White & Seymour, 2003; Kolokotrones et al., 2010; Savage et al., 2004; Spitz et al., 2018). Each method is represented by a color and different line type.

Table S4. Values of metabolic rates (kJ per day) calculated using different body-mass energy relationships using an average body length of 861 (±89) cm for female sperm whales in the Azores region, and respective assumptions of threshold foraging success rate for each method, considering an assimilation efficiency of 80%.

| **Method/ reference** | **Energy required (kJ/day)** | | | **Threshold foraging success rate (%)** | | | **Energy acquired (kJ/day) at threshold FSR** | |  |
| --- | --- | --- | --- | --- | --- | --- | --- | --- | --- |
|  | **mean** | **min** | **max** | |  | **mean** | | **Q1-Q3** | |
| Kleiber (1975) | 246692 | 197150 | 301951 | | 8 | 284985 | | 245007- 320295 | |
| Nagy et al. (1999) | 559650 | 449403 | 682064 | | 17 | 587717 | | 521155- 649789 | |
| White & Seymour (2003) | 87962 | 71998 | 105369 | | 4 | 134779 | | 109038- 156810 | |
| Savage et. Al (2004) | 191972 | 154017 | 234152 | | 6 | 212875 | | 178874- 242403 | |
| Kolokotrones et al. (2010) | 487832 | 363940 | 636686 | | 14 | 496048 | | 439263- 548377 | |
| ADMR, Spitz et al. (2018) | 493539 | 394425 | 604091 | | 14 | 496048 | | 439263- 548377 | |

## Prey structure scenarios

The potential energy acquired per capture attempt ($E_{PCA}$, kJ) was modelled as a log-normal distribution (Czapanskiy et al., 2021). The log normal distribution is parameterized in terms of location $\mu$ and scale $\sigma$ parameters, which are the mean and variance of the variable’s natural logarithm (e.g., of the transformed data) (Table S5). We will use $m$ and $s$ to represent the mean and standard deviation of the data (Table S5). The distribution for $E_{PCA}$ was described using sperm whales’ prey weight ($W_{prey}$; g) and energy density data ($ED$; kJ/g) (Goldbogen et al., 2019) (Table S2). Here, the location and scale parameters of log-normal distribution of $E_{PCA}$ were calculated from $\log(W_{prey}*ED)$ and weighted by the proportional contribution of each prey item in the diet (%). The baseline value for the variance of $E_{PCA}$ calculated from these historical diet records is assumed to represent the current variability in energy acquisition between prey capture attempts (green dashed curve, Fig. S2). In the model, changes in prey structure were reproduced by manipulating the baseline modelled distribution of $E_{PCA}$, particularly decreasing the mean ($m$) for prey size scenarios and decreasing the variance ($s^{2}$) for the variability scenarios. The weight in the tail of the distribution means whales have opportunities for feeding events with extremely high energetic content (Fig. S2). For the prey size scenarios, we left variance unchanged and decreased $m$ by 15% and 30%, to simulate a moderate and worst-case scenario respectively (orange dashed and blue dotted curves on Fig. S2, respectively). As for the scenarios representing changes in energy variability, we reduced the variance $s^{2}$by 30% and 50% (gold two dashed and pink dot dashed curves on Fig. S2, respectively) corresponding to moderate and worst-case scenarios respectively. Here, there is less variability, thus negating whales the possibility of feeding events with extremely high (or low) energetic content. In these scenarios, we left the mode unchanged, which did permit for a decrease in the mean. We chose this approach to make sure the scenario is biologically realistic, mimicking a situation in which large aggregations of prey are no longer available, requiring the whales to forage more constantly on patches that do not provide as much energy. Changing variance removes the extremely energy rich (and poor) patches observed in the neutral scenario, but also results in the mode shifting to the right if we maintain a constant mean. Thus, it would represent that the whales in these variance-reduction scenarios encounter higher energy patches more frequently than those in neutral scenario, which is counter to the intended biological situation.

Table S5. Parameters associated with log-normal distribution including a description of which parameters changed or remained the same for prey size and prey variability simulated scenarios.

| **Parameters** | **Abbreviation** | **Equation; Formula** | **Prey size** | **Prey variability** |
| --- | --- | --- | --- | --- |
| Location | $\mu$ | $\mu=\log m-\frac{log(1+{cv}^{2})}{2}$ | Changed | Changed |
| Scale | $\sigma$ | $\sigma=\sqrt{\log\left( 1+{cv}^{2} \right)}$ | Changed | Changed |
| Mean | $m$ | $m=e^{\mu+\frac{\sigma^{2}}{2}}$ | Decreased by 15% and 30% | Changed |
| Standard deviation | $s$ | $s=\sqrt{(e^{\sigma^{2}}-1)\times{(e}^{2\mu+\sigma^{2}})}$ | Remained the same | Decreased by 30% and 50% |
| Coefficient of variation | $cv$ | $cv=\frac{s}{m}=\sqrt{(e^{\sigma^{2}}-1)}$ | Changed | Changed |
| Mode |  | $Mode=e^{\mu-\sigma^{2}}$ | Changed | Remained the same |


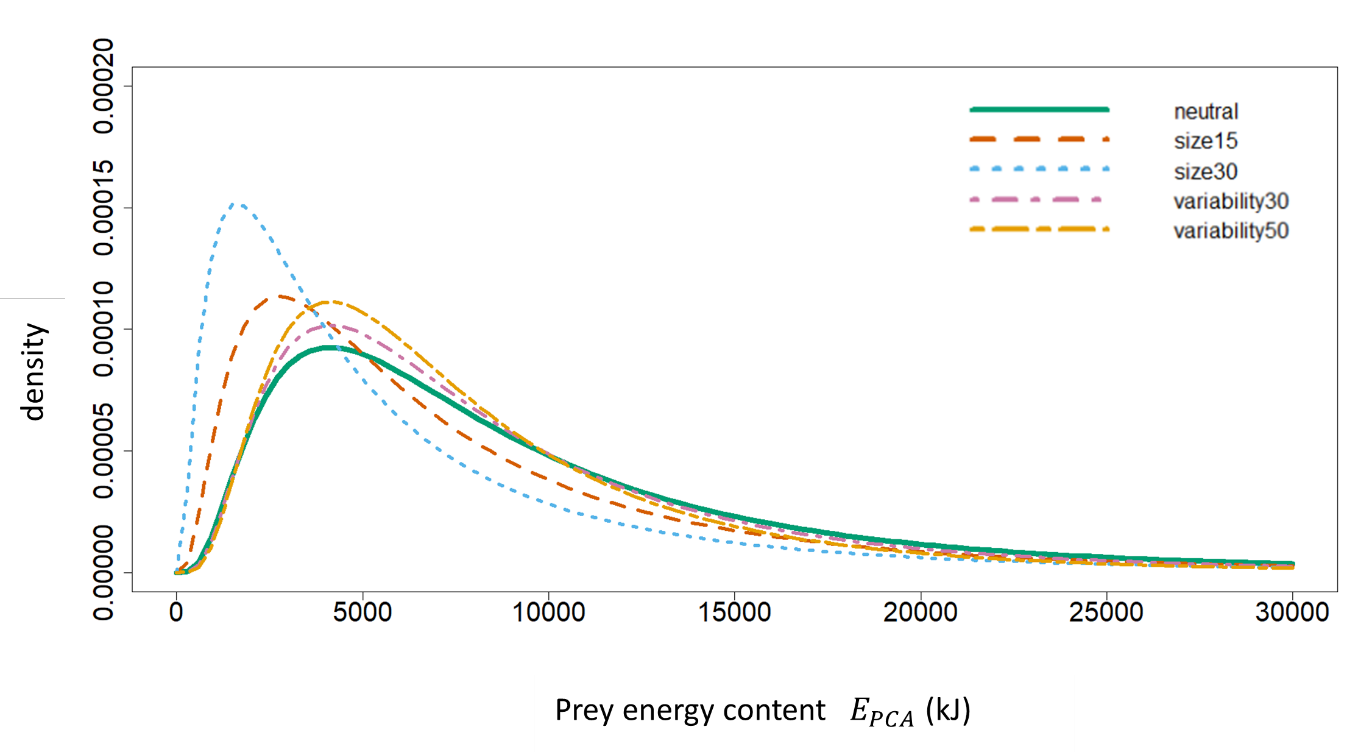
*Figure S2.* $E_{PCA}$ *density curves using different mean and variance values according to the scenarios simulated: baseline scenario in green solid line, prey size moderate 15% in orange dashed line, prey size worst 30% in blue dotted line, prey size variability moderate 30% in pink dot dashed line and prey size variability worst 50% in gold two dashed line.*

## Assimilation efficiency

Although the foraging success rate threshold of 14% varied little between the three assimilation efficiencies (74%, 80% and 95%) tested, at 14% foraging success rate, all assimilation efficiencies had similar observed outputs and patterns between them (Table S6; Figures S3 and S6).

Table S6. Results using assimilation efficiency of 74%, 80% and 95%: threshold foraging success rate (FSR, %) and correspondent energy acquired (kJ/day); proportion of compensation for each simulated scenario in relation to correspondent neutral scenario.

| **Assimilation efficiency** | **Simulated scenarios** | **Threshold Foraging Success Rate (FSR) %** | **Energy acquired (kJ/day) at correspondent threshold FSR** | **Y: proportion of compensation, % (at threshold FSR of the neutral case)** |
| --- | --- | --- | --- | --- |
| 74% | Neutral | 16 | 514369  (455962-567940) | - |
|  | Prey size 15% | 18 | 495501  (436042-548813) | 17 (2-29) |
|  | Prey size 30% | 22 | 504897  (442819-562087) | 43 (23-59) |
|  | Prey variability 30% | 18 | 529138  (472112-580854) | 9 (0-19) |
|  | Prey variability 50% | 19 | 507393  (456525-555220) | 19 (6-30) |
| 80% | Neutral | 14 | 496048  (439263-548377) | - |
|  | Prey size 15% | 17 | 499463  (439535-554142) | 21 (5-35) |
|  | Prey size 30% | 21 | 509579  (446091 567221) | 49 (27-67) |
|  | Prey variability 30% | 16 | 502837  (447433-552754) | 13 (0-23) |
|  | Prey variability 50% | 18 | 521318  (468646-571381) | 24 (10-35) |
| 95% | Neutral | 12 | 506756  (446922-560366) | - |
|  | Prey size 15% | 14 | 498892  (435182-555931) | 19 (3-33) |
|  | Prey size 30% | 18 | 524493  (453198-587406) | 46 (24-65) |
|  | Prey variability 30% | 13 | 497918  (442991-548731) | 10 (0-21) |
|  | Prey variability 50% | 15 | 509947  (455703-560595) | 21 (7-33) |


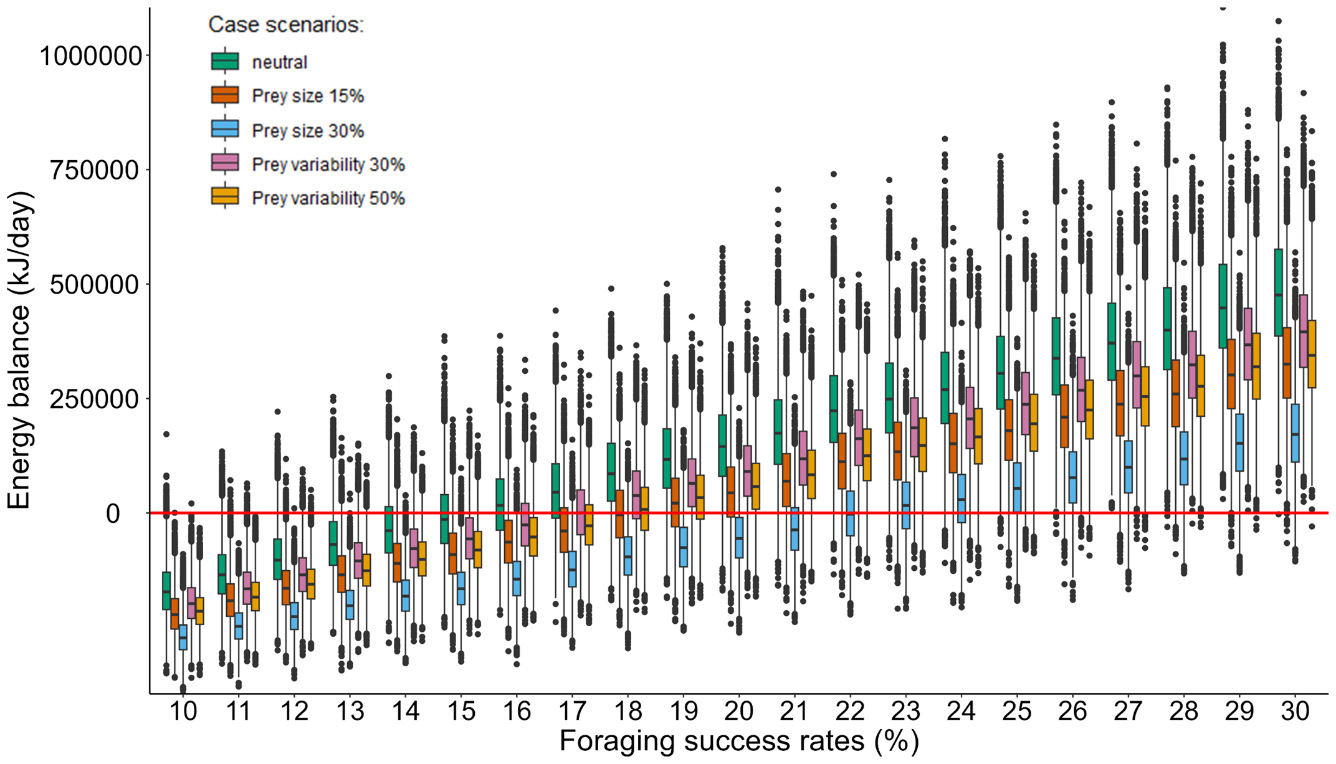


Figure S3. Energy balance per day as the difference between estimated energy acquisition per day relative to sperm whales’ estimated energy requirements over foraging success rate and assimilation efficiency of 74%, for all simulated scenarios. Case scenarios represented by colors – green: neutral-case (e.g., no changes); orange and blue: prey size moderate and worst with a decrease in prey’s size (energetic content) of 15% and 30%, respectively; purple and golden: prey variability moderate and worst with a decrease of 30% and 50% of energy availability, respectively. Reference red dashed line: average daily metabolic requirement (ADMR, kJ/day).


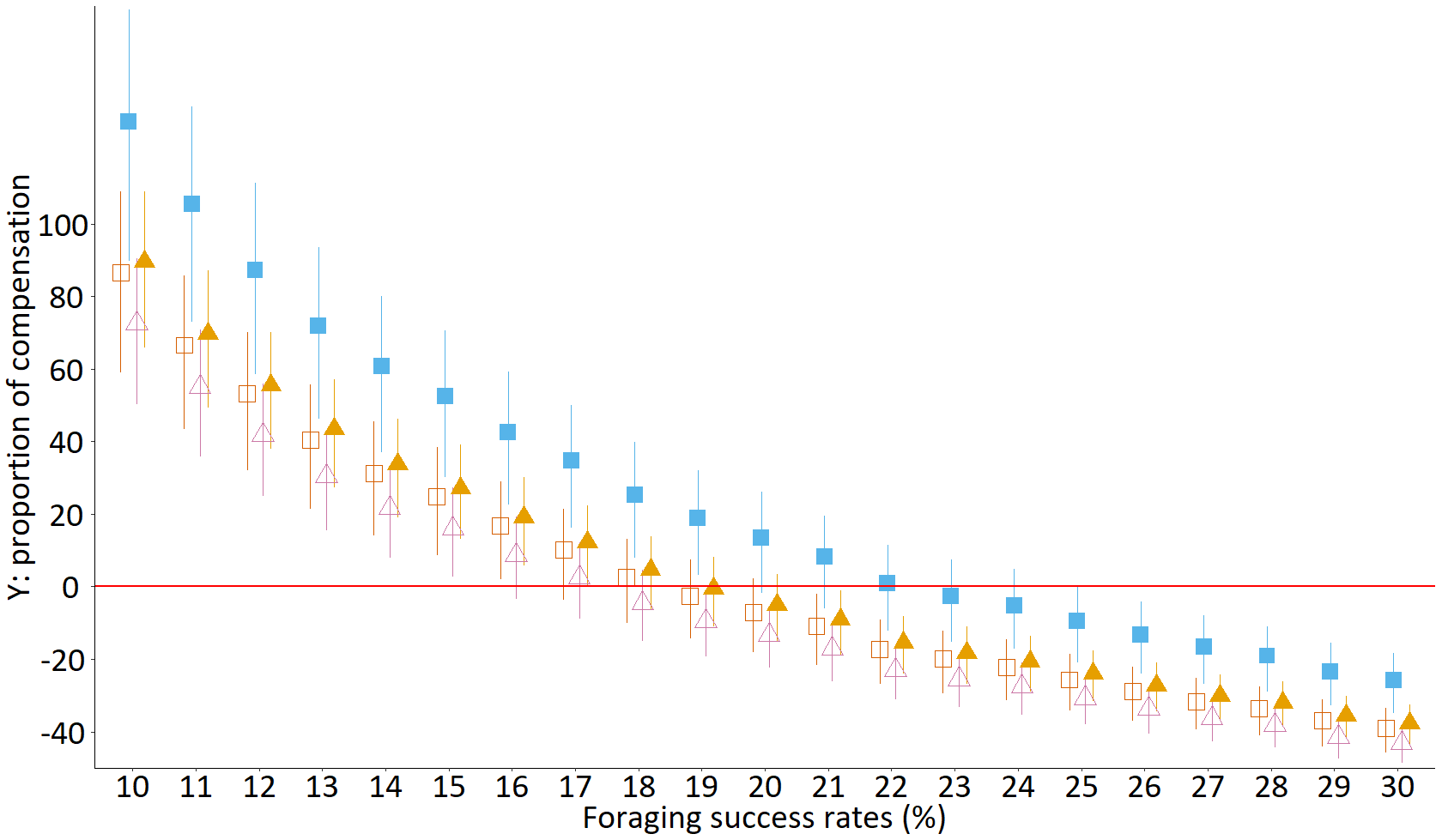


Figure S4. Compensation ratio (Y, the ratio of energy required to energy acquired per day, %) of tagged sperm whales in all simulated shift scenarios relative to their estimated energy requirements over foraging success and assimilation efficiency of 74%. Positive Y: insufficient energy acquired to meet mean energetic demands. Negative Y: surplus of energy gained as a proportion of mean energetic demands. Y=0: no compensation is needed. All four scenarios are represented, 15% decrease in prey size (empty orange square), 30% decrease in prey size (filled blue square), 30% decrease in variability (empty purple triangle) and 50% decrease in variability (filled gold triangle).


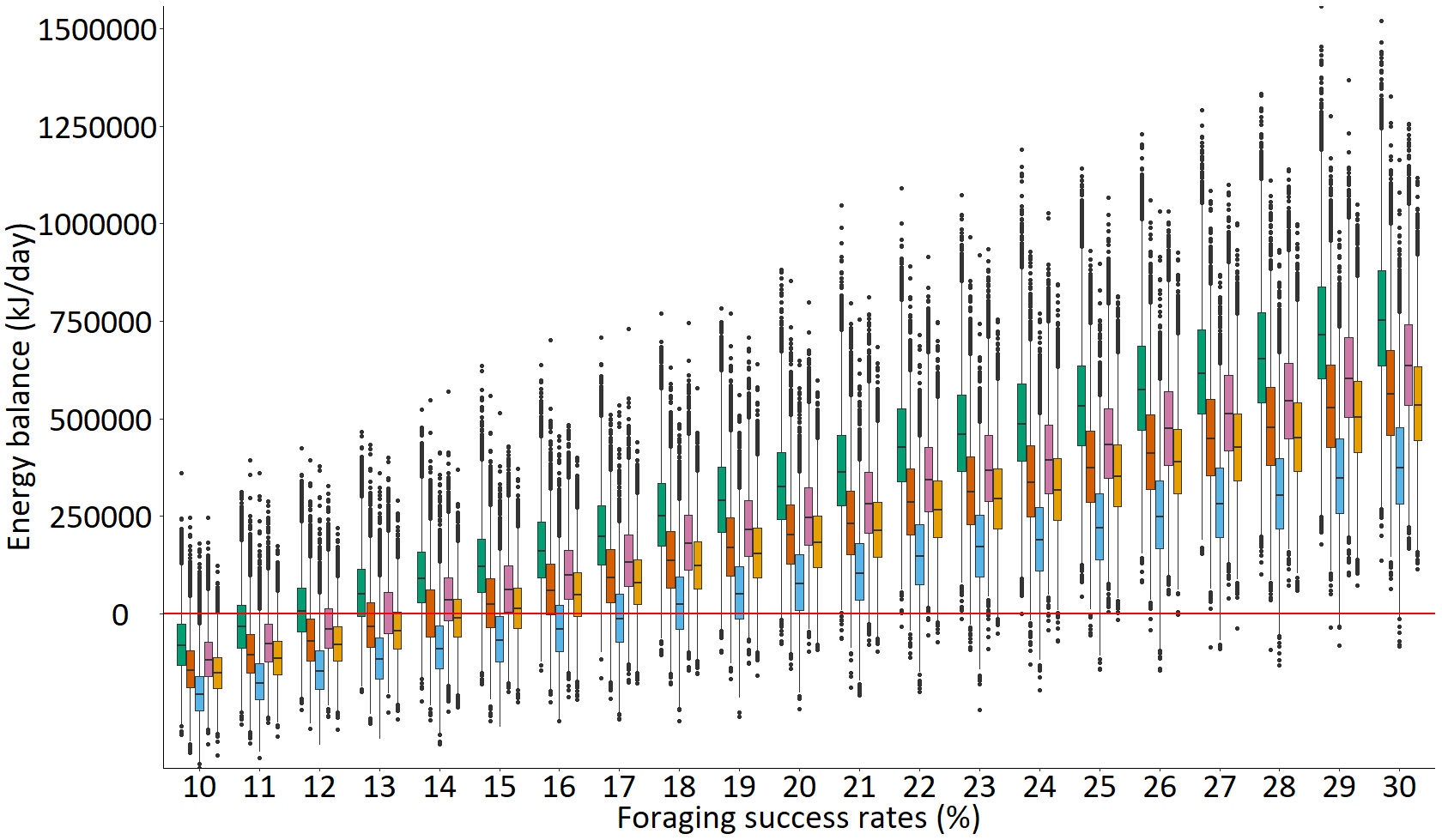


Figure S5. Energy balance per day as the difference between estimated energy acquisition per day relative to sperm whales’ estimated energy requirements over foraging success rate and assimilation efficiency of 95%, for all simulated scenarios. Case scenarios represented by colors - green: neutral-case (e.g., no changes); orange and blue: prey size moderate and worst with a decrease in prey’s size (energetic content) of 15% and 30%, respectively; purple and golden: prey variability moderate and worst with a decrease of 30% and 50% of energy variability, respectively. Reference red dashed line: average daily metabolic requirement (ADMR, kJ/day).


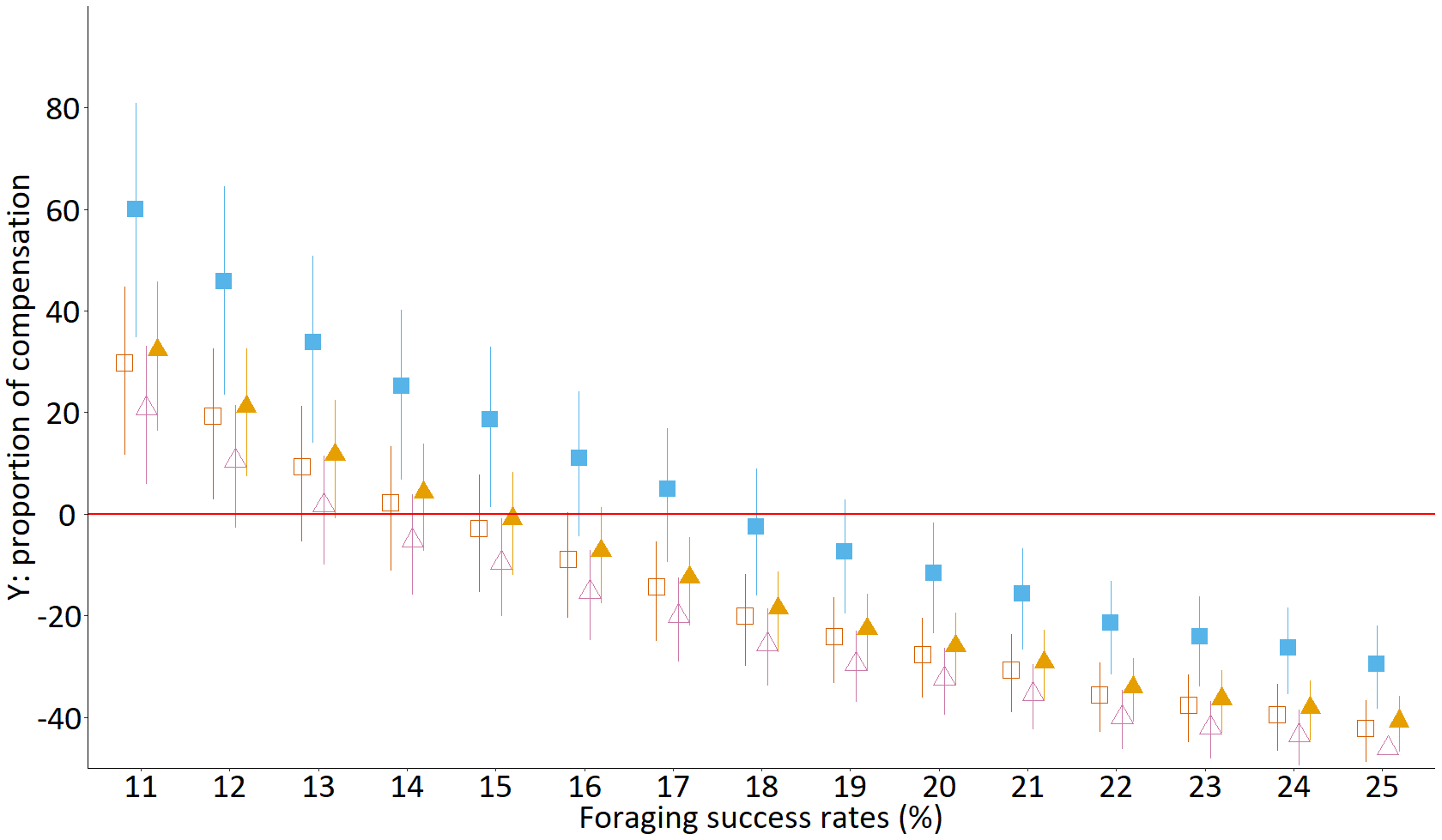


Figure S6. Compensation ratio (Y, the ratio of energy required to energy acquired per day, %) of tagged sperm whales in all simulated shift scenarios relative to their estimated energy requirements over foraging success rate and assimilation efficiency of 95%. Positive Y: insufficient energy acquired to meet mean energetic demands. Negative Y: surplus of energy gained as a proportion of mean energetic demands. Y=0: no compensation is needed. All four scenarios are represented, 15% decrease in prey size (empty orange square), 30% decrease in prey size (filled blue square), 30% decrease in variability (empty purple triangle) and 50% decrease in variability (filled gold triangle).

## References

Clarke, M. R. R., Martins, H. R., & Pascoe, P. (1993). The diet of sperm whales (Physeter macrocephalus Linnaeus 1758) off the Azores. *Philosophical Transactions - Royal Society of London, B*, *339*(1287), 67–82. https://doi.org/10.1098/rstb.1993.0005

Czapanskiy, M. F., Savoca, M. S., Gough, W. T., Segre, P. S., Wisniewska, D. M., Cade, D. E., & Goldbogen, J. A. (2021). Modelling short-term energetic costs of sonar disturbance to cetaceans using high-resolution foraging data. *Journal of Applied Ecology*, *58*(8), 1643–1657. https://doi.org/10.1111/1365-2664.13903

Foskolos, I., Koutouzi, N., Polychronidis, L., Alexiadou, P., & Frantzis, A. (2020). A taste for squid: the diet of sperm whales stranded in Greece, Eastern Mediterranean. *Deep-Sea Research Part I: Oceanographic Research Papers*, *155*(January 2019), 103164. https://doi.org/10.1016/j.dsr.2019.103164

Goldbogen, J. A., Cade, D. E., Wisniewska, D. M., Potvin, J., Segre, P. S., Savoca, M. S., Hazen, E. L., Czapanskiy, M. F., Kahane-Rapport, S. R., DeRuiter, S. L., Gero, S., Tønnesen, P., Gough, W. T., Hanson, M. B., Holt, M. M., Jensen, F. H., Simon, M., Stimpert, A. K., Arranz, P., … Pyenson, N. D. (2019). Why whales are big but not bigger: Physiological drivers and ecological limits in the age of ocean giants. *Science*, *366*(6471), 1367–1372. https://doi.org/10.1126/science.aax9044

Kolokotrones, T., Van Savage, Deeds, E. J., & Fontana, W. (2010). Curvature in metabolic scaling. *Nature*, *464*(7289), 753–756. https://doi.org/10.1038/nature08920

Lockyer, C. (1991). Body composition of the sperm whale, Physeter catodon , with special reference to the possible functions of fat depots. *Journal of the Marine Research Institute, Reykjavik.*, *12*(2), 1–25.

Max Kleiber. (1947). *Body size and metabolic rate*. *27*.

Nagy, K. A., Girard, I. A., & Brown, T. K. (1999). Energetics of free-ranging mammals, reptiles, and birds. *Annual Review of Nutrition*, *19*, 247–277. https://doi.org/10.1146/annurev.nutr.19.1.247

Savage, V. M., Gillooly, J. F., Woodruff, W. H., West, G. B., Allen, A. P., Enquist, B. J., & Brown, J. H. (2004). The predominance of quarter-power scaling in biology. *Functional Ecology*, *18*(2), 257–282. https://doi.org/10.1111/j.0269-8463.2004.00856.x

Spitz, J., Ridoux, V., Trites, A. W., Laran, S., & Authier, M. (2018). Prey consumption by cetaceans reveals the importance of energy-rich food webs in the Bay of Biscay. *Progress in Oceanography*, *166*, 148–158. https://doi.org/10.1016/j.pocean.2017.09.013

White, C. R., & Seymour, R. S. (2003). Mammalian basal metabolic rate is proportional to body mass2/3. *Proceedings of the National Academy of Sciences of the United States of America*, *100*(7), 4046–4049. https://doi.org/10.1073/pnas.0436428100
